# Efficacy and Safety of Faropenem in Bangladeshi Adults With Community-Acquired Pneumonia: A Randomized Controlled Trial

**DOI:** 10.7759/cureus.118941

**Published:** 2026-11-01

**Authors:** Khan Abul Kalam Azad, Quazi Tarikul Islam, H A M Nazmul Ahasan, Mohammad Zahiruddin, Mohammad Rafiqul Islam, Rubina Yasmin, Syed Ghulam Mogni Mowla, K M Ashik Elahi, Samia Tahnin Reza, Mohammed Alimur Reza

**Affiliations:** 1 Department of Medicine, Popular Medical College, Dhaka, BGD; 2 Department of Medicine, Shaheed Suhrawardy Medical College, Dhaka, BGD; 3 Department of Internal Medicine, LABAID Specialized Hospital, Dhaka, BGD; 4 Department of Medical Affairs, Beximco Pharmaceuticals PLC, Dhaka, BGD

**Keywords:** antimicrobial stewardship, bangladesh, clarithromycin, co-amoxiclav, community-acquired pneumonia, faropenem, randomized controlled trial

## Abstract

Background: Community-acquired pneumonia (CAP) causes substantial morbidity, and effective single-agent oral therapies that simplify treatment are needed. Faropenem, an oral penem with broad antibacterial activity, has not previously been compared with standard combination therapy for CAP. This study aimed to evaluate the efficacy and safety of oral faropenem, compared with co-amoxiclav plus clarithromycin, in adults with mild-to-moderate CAP who did not require hospitalization.

Materials and methods: In this open-label, randomized controlled trial conducted at two tertiary centers in Dhaka, Bangladesh, adults aged 18-65 years with mild-to-moderate CAP were randomly assigned in a 1:1 ratio to oral faropenem or combination therapy. Patients with severe pneumonia, recent hospitalization, or significant renal or hepatic impairment were excluded 200 mg three times daily or co-amoxiclav 625 mg three times daily plus clarithromycin 500 mg twice daily, each for 7 days with standard care. The primary efficacy outcome was clinical cure at days 10-14, and the primary safety outcome was withdrawal due to adverse events (ClinicalTrials.gov NCT06804096).

Results: Of 244 patients assessed, 129 were randomized (faropenem, 65; comparator, 64), and all completed follow-up. Clinical cure at days 10-14 was 98.5% with faropenem and 95.3% with the comparator (risk difference +3.1%, 95% confidence interval (CI) -2.8 to +9.1; relative risk 1.03, 95% CI 0.97-1.10). The clinical cure at day 3 was 84.6% versus 78.1%. Adverse events were infrequent (9.2% versus 12.5% in the faropenem and comparator arms, respectively), with no serious adverse events and no treatment withdrawals; altered taste occurred only in the comparator arm. Hepatic and renal parameters showed no clinically meaningful change in either arm.

Conclusions: In adults with mild-to-moderate CAP, oral faropenem was non-inferior to co-amoxiclav and clarithromycin. As a single-agent oral therapy, it may offer an alternative when narrower-spectrum first-line regimens are unsuitable. However, larger, blinded, microbiologically well-characterized trials are needed to establish pathogen-specific efficacy. These findings should not be extrapolated to severe or hospitalized CAP or to patients with significant renal or hepatic impairment.

## Introduction

Community-acquired pneumonia (CAP) is among the most common serious infections in adult practice and a leading infectious cause of death worldwide, including in Bangladesh. Lower respiratory tract infections impose a substantial and, in many regions, increasing burden, which is further complicated by antimicrobial resistance [1]. This burden falls disproportionately on low- and middle-income countries; in Bangladesh, CAP is a frequent cause of adult morbidity, and concerning rates of antimicrobial resistance have been reported among respiratory and invasive bacterial isolates [2,3].

Empirical therapy is directed at the principal bacterial pathogens of CAP [4,5]. Current guidance from the American Thoracic Society and the Infectious Diseases Society of America recommends an aminopenicillin, doxycycline, or a macrolide for outpatients without comorbidities, and a beta-lactam combined with a macrolide, or a respiratory fluoroquinolone, for those with comorbidities [4]; the beta-lactam plus macrolide combination has been examined in systematic reviews and meta-analyses in CAP [6]. However, increasing resistance to commonly used beta-lactams, macrolides, and fluoroquinolones, together with a limited pipeline of genuinely new antibacterial agents, has renewed interest in effective, well-tolerated oral options, particularly in resource-limited settings where parenteral therapy and hospitalization are not always feasible.

Faropenem is an oral penem-class beta-lactam with broad activity against Gram-positive and Gram-negative respiratory pathogens, including *Streptococcus pneumoniae*, *Haemophilus influenzae*, and *Moraxella catarrhalis*, and relative stability against many beta-lactamases [5,7]. It is licensed primarily in Japan and India and has been evaluated for respiratory tract infections; randomized trials in acute bacterial sinusitis found it comparable to cefuroxime axetil [8,9]. Robust CAP-specific evidence, particularly among adult populations in South Asian countries such as Bangladesh, remains limited. At the same time, because faropenem is structurally related to carbapenems, its expanding use has raised antimicrobial stewardship concerns about potential selection for carbapenem cross-resistance [10], underscoring the need to rigorously evaluate both its efficacy and its appropriate positioning.

We therefore conducted a randomized controlled trial to evaluate the efficacy and safety of oral faropenem compared with co-amoxiclav plus clarithromycin in Bangladeshi adults with mild-to-moderate CAP. The primary objective was to compare clinical cure rates between the two regimens; secondary objectives included early clinical response, all-cause mortality, hospitalization and intensive care requirements, the frequency of adverse events, and changes in hepatic and renal function.

## Materials and methods

Study design and setting

This prospective, open-label, randomized controlled, phase 4 non-inferiority trial (ClinicalTrials.gov NCT06804096) was conducted at Popular Medical College Hospital and Shaheed Suhrawardy Medical College Hospital, Dhaka, Bangladesh, with recruitment from February 20, 2025 to January 12, 2026. The trial was reported in accordance with the Consolidated Standards of Reporting Trials (CONSORT) statement [11]. The study was conducted in accordance with the Declaration of Helsinki. Ethical approval was obtained from the Ethics Committee of Popular Medical College (approval number: PMC/IEthicalrb/2024/044). Written informed consent was obtained from all participants before enrollment.

Participants

Eligible participants were adults aged 18-65 years with CAP. Eligibility required an acute illness lasting seven days or less, characterized by new or worsening symptoms of a lower respiratory tract infection, including fever (>38.0°C (100.4°F)), shortness of breath, new or increased cough with or without sputum production, or chest pain, together with radiographically documented pneumonia.

Key exclusion criteria were severe pneumonia, judged by the treating investigator at screening on clinical and radiological assessment, specifically hypothermia, tachycardia, or respiratory distress (dyspnea at rest), rather than by a formal severity score; suspected viral pneumonia, for example, bilateral patchy opacities, or suspected nosocomial or aspiration pneumonia; known hypersensitivity or contraindication to any study drug; receipt of an antibiotic within the preceding 48 hours; hospitalization within the previous 28 days; pregnancy or lactation; renal impairment (screening estimated glomerular filtration rate (eGFR) <30 mL/min/1.73 m²); significant hepatic impairment (alanine aminotransferase (ALT) >3 times the upper limit of normal); serious immunocompromising conditions such as HIV infection or malignancy; use of systemic corticosteroids (≥20 mg/day prednisolone equivalent); chemotherapy or anticancer therapy during the trial or within the preceding six months; epilepsy, other central nervous system disorders, or uncontrolled psychiatric illness; or any condition rendering the patient unsuitable in the investigator’s judgment.

Randomization and blinding

After confirmation of eligibility and written informed consent, participants were randomly assigned in a 1:1 ratio to faropenem or to co-amoxiclav plus clarithromycin. The allocation sequence was generated from a random number table using permuted blocks of four, stratified by investigator, by an independent person who had no role in participant enrollment, treatment assignment, outcome assessment, or data analysis. Allocation was concealed using sequentially numbered, sealed envelopes prepared in that sequence; each envelope was opened only after a participant was confirmed eligible and had provided written informed consent. Participants were enrolled by the site investigators, who also assigned the allocated treatment. The trial was open label; neither participants nor investigators were masked to treatment allocation after assignment.

Interventions

Participants in the faropenem arm received oral faropenem 200 mg three times daily for seven days. Participants in the comparator arm received oral co-amoxiclav 625 mg three times daily plus clarithromycin 500 mg twice daily for seven days. Both arms received standard supportive care. Study medication was supplied by the sponsor and taken with water, with or without food. The study prohibited concomitant medications with clinically important interactions with the study drugs and recorded all other treatments taken during the study. All participants received the comparator as a single fixed regimen, regardless of comorbidity status; at the same doses, this combination corresponds to the beta-lactam plus macrolide regimen recommended by the 2019 ATS/IDSA guideline for outpatients with comorbidities [4].

Outcomes

The primary efficacy outcome was the clinical cure rate at days 10-14, compared between the two treatment arms. Clinical cure was defined as clinically significant improvement in signs and symptoms, specifically resolution or improvement in at least three of six clinical features (fever, shortness of breath, chest pain, cough, sputum or mucus production, and overall sense of well-being) as judged by the treating physician, with no worsening of any feature, together with the absence of new-onset symptoms, complications, or any need for further antimicrobial therapy. Each feature was recorded by the treating physician at every assessment as absent or, if present, as improved, unchanged, or worse relative to baseline; no formal severity scale was used. The primary safety outcome was the proportion of patients withdrawn from the study because of adverse events, compared between the two arms and assessed at days 10-14.

Secondary efficacy outcomes were early clinical response, all-cause mortality, the proportion of patients requiring hospitalization, and the proportion requiring intensive care unit (ICU) support. Early clinical response was defined as improvement in at least one symptom within 72 ± 24 hours (days 2-4) of the first dose of study drug.

The secondary safety outcome was the frequency of adverse events and serious adverse events, compared between the two arms through day 28. Safety was evaluated by continuous monitoring and recording of all adverse events and serious adverse events throughout the study period. Additionally, routine laboratory safety monitoring was performed: ALT, creatinine, urea, and eGFR, calculated using the 2021 Chronic Kidney Disease Epidemiology Collaboration (CKD-EPI) creatinine equation [12], were measured at baseline and at the end of follow-up (day 28), and treatment-emergent laboratory abnormalities were recorded as part of the safety analysis.

Study procedures and follow-up

Eligible patients provided written informed consent and were randomly assigned to one of two treatment arms. At baseline, each participant underwent a full clinical evaluation, including history, physical examination, and assessment of the six target signs and symptoms (fever, shortness of breath, chest pain, cough, sputum or mucus production, and sense of well-being); body temperature was measured orally. Chest radiography confirmed the diagnosis of CAP. Baseline laboratory investigations included a complete blood count with total leukocyte count (TLC), ALT, creatinine, urea, and eGFR. No microbiological or serological sampling was performed.

Participants were followed at three scheduled post-baseline visits. At the first follow-up (day 3), conducted by telephone, target signs and symptoms were reassessed to evaluate early clinical response. At the second follow-up visit (days 10-14), signs and symptoms were reassessed to determine the primary efficacy outcome of clinical cure following treatment completion. At the final visit (day 28, end of follow-up), clinical status was reassessed, and the baseline laboratory investigations (TLC, ALT, creatinine, urea, and eGFR) were repeated. The need for hospitalization or ICU support at any time through day 28 was recorded. Adverse events and serious adverse events were monitored and recorded continuously throughout the study period, from the first dose of study drug through the end of follow-up.

Sample size

The sample size was calculated for a one-sided non-inferiority comparison of two independent proportions on the risk-difference scale, using the sample size calculator of the Department of Quantitative Health Sciences, Cleveland Clinic. Assuming clinical cure rates of 91.5% in the faropenem arm [13] and 81.6% in the comparator arm [14], a one-sided α of 0.025, 90% power, 1:1 allocation, and a prespecified non-inferiority margin of 10 percentage points on the risk-difference scale [15], a minimum evaluable sample of 122 participants (61 per arm) was required. To account for potential dropout or non-evaluability, the protocol specified enrollment of up to 160 participants (80 per arm), consistent with the target sample size recorded at trial registration. A total of 129 participants were enrolled and randomized (65 to the faropenem group and 64 to the comparator group). No participant was lost to follow-up, and primary outcome data were available for all randomized participants; therefore, all 129 were evaluable and included in the primary analysis, exceeding the minimum required evaluable sample of 122.

Statistical analysis

Data were recorded on structured case report forms by trained research staff and checked for completeness and consistency before analysis. Analyses were performed on all randomized participants (full analysis set). The distribution of continuous variables was assessed for normality; continuous variables were summarized as mean ± standard deviation or median (interquartile range (IQR)), as appropriate, and categorical variables as frequencies and percentages. Baseline characteristics are presented descriptively without significance testing. Categorical outcomes were compared between arms using the Fisher exact test; within-participant changes in hepatic and renal parameters were compared between arms using the Mann-Whitney U test.

For the primary outcome, the treatment effect was expressed as the absolute risk difference and the relative risk, each with a two-sided 95% confidence interval (CI). Non-inferiority of faropenem was concluded if the lower limit of the two-sided 95% CI for the risk difference excluded the prespecified margin of -10 percentage points, which corresponds to a one-sided test at an α of 0.025. Because follow-up was complete, no imputation for missing data was required. A two-sided p-value < 0.05 was considered statistically significant for all other comparisons. Analyses were conducted using SPSS Statistics version 25.0 (IBM Corp. Released 2017. IBM SPSS Statistics for Windows, Version 25.0. Armonk, NY).

## Results

Participant flow and baseline characteristics

A total of 244 patients with suspected CAP were assessed for eligibility, of whom 115 were excluded (68 did not meet the inclusion criteria, 33 declined to participate, and 14 for other reasons). The remaining 129 patients were randomized to faropenem (n = 65) or co-amoxiclav plus clarithromycin (n = 64). All randomized participants received their allocated treatment, completed follow-up through day 28, and were included in the analysis, with no losses to follow-up and no discontinuations (Figure 1).

**Figure 1:**
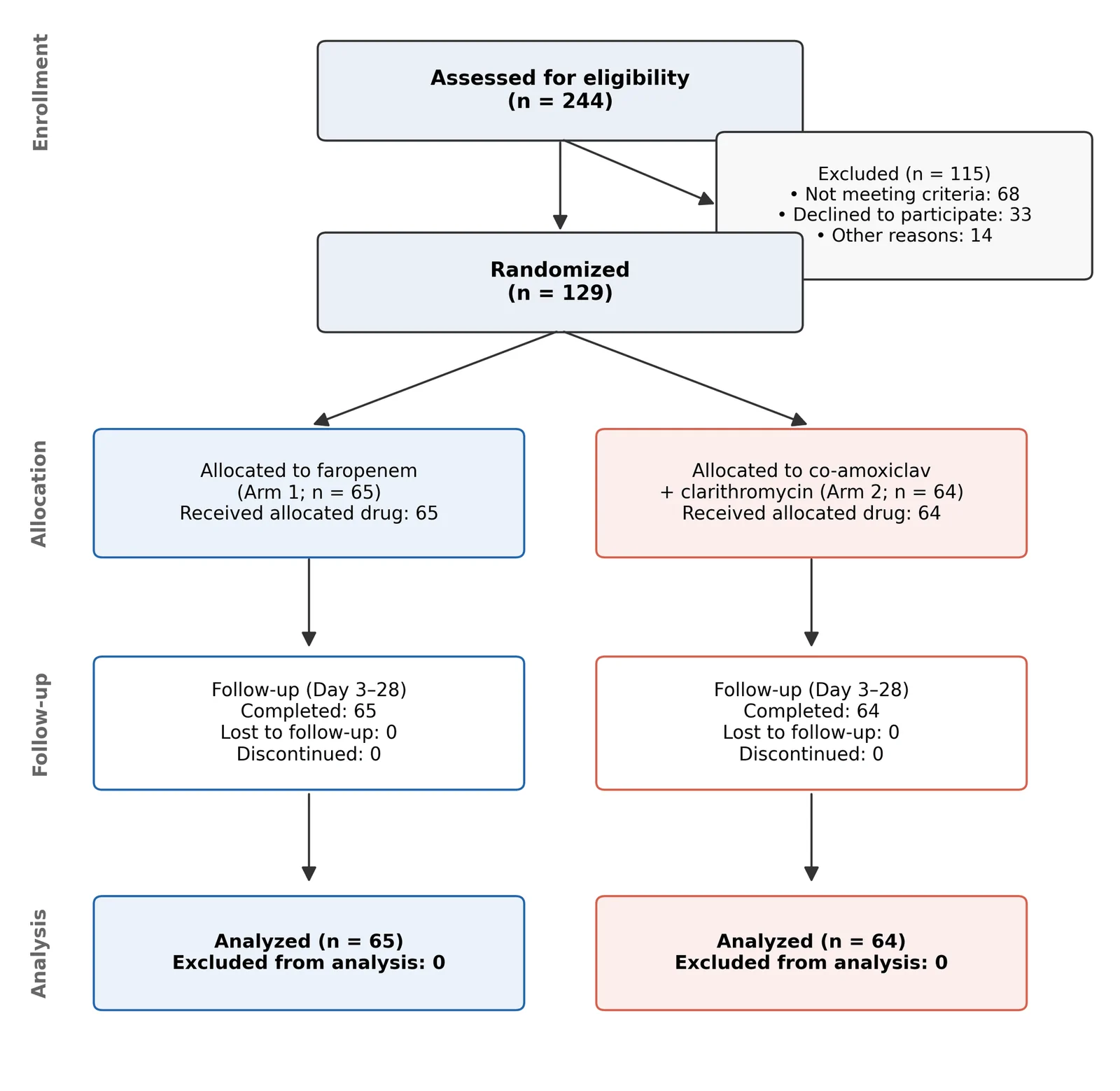
CONSORT participant flow diagram for a randomized comparison of faropenem versus co-amoxiclav plus clarithromycin in CAP (N = 129) CONSORT: Consolidated Standards of Reporting Trials, CAP: community-acquired pneumonia

Baseline demographic, clinical, and laboratory characteristics were comparable between the two arms (Table 1). The median age was 55.0 years (IQR, 39.0-60.0) in the faropenem arm and 52.5 years (IQR, 39.8-59.0) in the comparator arm, and 62 participants (48.1%) were male. The mean body mass index was 27.0 ± 3.6 kg/m² and 27.1 ± 3.7 kg/m², respectively. At least one comorbidity was present in 83 participants (64.3%), most commonly hypertension (40.3%) and diabetes mellitus (40.3%). Cough and sputum production were universal (100%), while fever (94.6%) and shortness of breath (86.8%) were the next most frequent presenting features; chest pain was reported by only one participant (0.8%). Chest radiography showed left-lung involvement in 60 participants (46.5%), right-lung involvement in 35 (27.1%), and bilateral involvement in 34 (26.4%). Baseline laboratory values were comparable across arms. All 129 participants had at least three of the six target signs and symptoms present at baseline.

**Table 1:** Baseline characteristics by treatment arm Data are presented as median (IQR), mean ± SD, or n (%). Baseline characteristics are presented descriptively; no significance testing was performed. eGFR: estimated glomerular filtration rate, ALT: alanine aminotransferase, IQR: interquartile range, SD: standard deviation

Characteristic	Faropenem (n = 65)	Co-amoxiclav + clarithromycin (n = 64)
Demographics		
Age, years	55.0 (39.0-60.0)	52.5 (39.8-59.0)
Sex, male	27 (41.5)	35 (54.7)
Body mass index, kg/m²	27.0 ± 3.6	27.1 ± 3.7
Comorbidities		
Any comorbidity	42 (64.6)	41 (64.1)
Hypertension	26 (40.0)	26 (40.6)
Diabetes mellitus	28 (43.1)	24 (37.5)
Presenting features		
Fever	60 (92.3)	62 (96.9)
Shortness of breath	57 (87.7)	55 (85.9)
Cough	65 (100.0)	64 (100.0)
Sputum production	65 (100.0)	64 (100.0)
Chest pain	1 (1.5)	0 (0.0)
Vital signs		
Temperature, °F	100.5 (100.0-101.5)	101.0 (100.0-101.5)
Respiratory rate, breaths/min	18.0 (16.0-20.0)	17.5 (16.0-20.0)
Pulse, beats/min	80 (76-92)	84 (76-89)
Systolic blood pressure, mmHg	120 (110-130)	125 (120-140)
Chest radiographic involvement		
Right lung	15 (23.1)	20 (31.2)
Left lung	32 (49.2)	28 (43.8)
Bilateral	18 (27.7)	16 (25.0)
Baseline investigations		
Total leukocytes, ×10⁹/L	12.3 (9.9-13.6)	12.1 (10.5-12.9)
Hemoglobin, g/dL	13.1 (12.2-13.7)	12.8 (12.0-13.9)
Neutrophils, %	69.0 (64.0-81.0)	70.0 (65.0-77.5)
Lymphocytes, %	24.0 (16.0-31.0)	23.5 (18.2-28.0)
ALT, U/L	29.0 (21.0-34.0)	27.5 (20.0-36.0)
Serum creatinine, mg/dL	0.9 (0.8-1.1)	1.0 (0.8-1.1)
Blood urea, mg/dL	23 (19-27)	22 (17-29)
eGFR, mL/min/1.73 m²	87.8 (70.0-105.1)	89.5 (76.3-101.7)

Efficacy outcomes

A clinical cure at days 10-14 (primary outcome) was achieved by 125 of 129 participants (96.9%) overall. Cure rates were high and similar between the two treatment arms: 64 of 65 (98.5%) with faropenem and 61 of 64 (95.3%) with co-amoxiclav plus clarithromycin (p = 0.37) (Table 2). The corresponding risk difference was +3.1% (95% CI, -2.8 to +9.1), and the relative risk was 1.03 (95% CI, 0.97-1.10), indicating comparable efficacy between the two regimens.

**Table 2:** Efficacy outcomes and treatment effect estimates by treatment arm Data are n (%). Between-arm differences are summarized as risk differences and relative risks with 95% CIs; the risk difference is the faropenem proportion minus the comparator proportion, so positive values favor faropenem. No effect estimate is shown (-) for early clinical response, which occurred in every participant in both arms. Corresponding p-values from the Fisher exact test are reported in the text. CI: confidence interval

Outcome	Faropenem (n = 65)	Co-amoxiclav + clarithromycin (n = 64)	Risk difference (95% CI)	Relative risk (95% CI)
Primary efficacy outcome				
Clinical cure at days 10-14	64 (98.5)	61 (95.3)	+3.1% (-2.8 to +9.1)	1.03 (0.97-1.10)
Secondary efficacy outcomes				
Early clinical response at day 3	65 (100.0)	64 (100.0)	-	-
Clinical cure at day 3	55 (84.6)	50 (78.1)	+6.5% (-6.9 to +19.9)	1.08 (0.92-1.28)

Early clinical response at day 3 occurred in all participants in both arms (100%). Clinical cure at day 3, assessed by telephone, was achieved by 105 of 129 participants (81.4%) overall: 55 of 65 (84.6%) with faropenem and 50 of 64 (78.1%) with the comparator (p = 0.37; risk difference +6.5%, 95% CI, -6.9 to +19.9; relative risk 1.08, 95% CI, 0.92-1.28) (Table 2). No deaths, hospitalizations, or intensive care admissions occurred in either arm during the 28-day follow-up period.

Safety outcomes

No participant in either arm was withdrawn from the study because of an adverse event (primary safety outcome) (Table 3). Treatment-emergent adverse events were reported in 14 participants (10.9%) overall, with a similar frequency in the faropenem arm (6 of 65; 9.2%) and the comparator arm (8 of 64; 12.5%). The most frequently reported events were diarrhea (3.1%) and altered taste (3.1%), followed by vomiting (2.3%) and abdominal pain (2.3%); altered taste occurred only in the comparator arm (0% versus 6.2%). No serious adverse events were recorded, and all reported events were non-serious and self-limiting.

**Table 3:** Safety and adverse event outcomes by treatment arm Data are n (%). Participants could report more than one event, so the sum of individual events may exceed the number of participants reporting any adverse event.

Outcome	Faropenem (n = 65)	Co-amoxiclav + clarithromycin (n = 64)
Primary safety outcome		
Withdrawal due to adverse event	0 (0.0)	0 (0.0)
Adverse events (secondary safety outcome)		
Any adverse event	6 (9.2)	8 (12.5)
Vomiting	2 (3.1)	1 (1.6)
Abdominal pain	2 (3.1)	1 (1.6)
Diarrhea	2 (3.1)	2 (3.1)
Altered taste	0 (0.0)	4 (6.2)
Headache	0 (0.0)	1 (1.6)

Changes in hepatic and renal parameters from baseline to end of follow-up were small and not clinically meaningful in either arm (Table 4). Median ALT, serum creatinine, blood urea, and eGFR values were essentially unchanged, with no significant between-group differences.

**Table 4:** Change in liver and kidney function from baseline to end of follow-up by treatment arm Data are median (IQR). The p-value reflects the between-arm comparison of within-participant change from baseline to day 28, tested with the Mann-Whitney U test; U is the smaller of the two Mann-Whitney U statistics. eGFR: estimated glomerular filtration rate, ALT: alanine aminotransferase, IQR: interquartile range

Parameter	Group	Baseline	Day 28	Test Statistic	p-value
Liver function					
ALT, U/L	Faropenem	29.0 (21.0-34.0)	28.0 (21.0-35.0)	U = 1970.5	0.6
	Co-amoxiclav + clarithromycin	27.5 (20.0-36.0)	29.0 (21.8-34.0)		
Kidney function					
Serum creatinine, mg/dL	Faropenem	0.9 (0.8-1.1)	0.9 (0.8-1.0)	U = 1999.0	0.7
	Co-amoxiclav + clarithromycin	1.0 (0.8-1.1)	0.9 (0.8-1.1)		
Blood urea, mg/dL	Faropenem	23.0 (19.0-27.0)	22.0 (19.0-27.0)	U = 1874.0	0.33
	Co-amoxiclav + clarithromycin	22.0 (17.0-29.0)	22.0 (18.0-28.0)		
eGFR, mL/min/1.73 m²	Faropenem	87.8 (70.0-105.1)	88.9 (70.6-103.7)	U = 1987.0	0.66
	Co-amoxiclav + clarithromycin	89.5 (76.3-101.7)	87.9 (75.7-104.1)		

## Discussion

In this multicenter, open-label randomized controlled trial of adults with mild-to-moderate CAP, oral faropenem achieved a days 10-14 clinical cure rate of 98.5%, compared with 95.3% for co-amoxiclav plus clarithromycin. Because the lower bound of the 95% CI for the risk difference (-2.8%) lay well above the prespecified -10% non-inferiority margin, faropenem met the criterion for non-inferiority for days 10-14 clinical cure; the confidence interval included zero, indicating that the numerically higher cure rate observed with faropenem should be interpreted as comparable efficacy rather than evidence of superiority. The secondary endpoints reinforced this interpretation: every participant in both arms had an early clinical response, and clinical cure was already attained by day 3 in 84.6% of faropenem and 78.1% of comparator recipients (risk difference +6.5%; 95% CI, -6.9 to +19.9). Both treatments were well tolerated, with no serious adverse events, no withdrawals due to adverse events, and no clinically meaningful changes in hepatic or renal function over the study period.

The high cure rate observed with faropenem is consistent with its earlier phase III experience in CAP. In a multinational, double-blind randomized trial, oral faropenem medoxomil achieved clinical cure in 91.5% of clinically evaluable patients, comparable to 88.4% with high-dose amoxicillin [13]; separate randomized CAP trials likewise found faropenem broadly equivalent to co-amoxiclav 625 mg three times daily (86.4% versus 88.1%) and to cefpodoxime (approximately 89.5% versus 88.6%) [7]. Against this background, the cure rates of approximately 95-98% observed in the present study fall at the upper end of the previously reported range. Newer oral agents, including omadacycline, lefamulin, and nemonoxacin, have demonstrated clinical response rates predominantly in the mid-80s to low-90s [15-18]. Our somewhat higher rates most plausibly reflect enrollment restricted to mild-to-moderate, non-severe disease, complete follow-up without attrition, and an open-label, clinician-assessed cure endpoint. The efficacy observed with single-agent faropenem is biologically plausible given its activity against the common typical respiratory pathogens and its stability against many beta-lactamases [5,7]. Unlike the comparator, faropenem provides no coverage against atypical pathogens, for which a macrolide was included in the comparator regimen [4]. The finding of non-inferiority may suggest that atypical pathogens were uncommon or contributed minimally to mild-to-moderate disease; however, neither possibility can be verified without microbiological testing. Pathogen-specific efficacy, the contribution of atypical organisms, and microbiological equivalence therefore remain undetermined.

The cohort reflected several characteristics of adults with CAP in South Asia, including a median age of 53 years, a nearly equal sex distribution, and a high burden of comorbidities. Hypertension and diabetes mellitus were each present in approximately 40% of participants, consistent with the increasing burden of noncommunicable diseases in Bangladesh [3]. These characteristics may support the applicability of the findings to similar patient populations [3]. By design, patients with significant renal or hepatic impairment were excluded, so baseline renal and hepatic function was largely preserved (median eGFR 87.8 and 89.5 mL/min/1.73 m²; median ALT 29.0 and 27.5 U/L), though participants with milder reductions in eGFR or transaminase elevations were eligible. This provided a stable background against which treatment-emergent hepatic and renal changes could be assessed. The two arms were well matched at baseline across all measured demographic, clinical, and laboratory characteristics.

The safety profile observed here is consistent with the beta-lactam and macrolide classes studied and with the favorable tolerability previously reported for faropenem [7]. Adverse events were infrequent (10.9% overall), predominantly gastrointestinal, and altered taste occurred only in the comparator arm, in keeping with the well-recognized dysgeusia associated with clarithromycin. Laboratory measures reinforced this impression: median hepatic and renal indices were essentially unchanged from baseline to the end of follow-up, with no significant between-group differences (Table 4). The absence of serious adverse events, treatment discontinuations, and any significant hepatic or renal signal supports the short-term tolerability of both regimens. However, the absence of a safety signal in a trial of this size does not establish safety. The study was neither designed nor powered to detect uncommon or delayed adverse events, which therefore cannot be excluded.

Beyond comparable efficacy, the two regimens differ in complexity. Faropenem was administered as a single agent three times daily, whereas the comparator regimen comprised two agents administered three and two times daily, respectively. This may reduce pill burden and avoid the cytochrome P450 3A4 interactions and QT-prolongation warnings of clarithromycin [19]. However, this convenience must be distinguished from the antimicrobial spectrum. As a beta-lactam, faropenem lacks activity against atypical pathogens such as Mycoplasma pneumoniae. Without etiological testing, this trial cannot determine whether atypical pathogens were present or adequately treated.

As a broad-spectrum penem, faropenem raises antimicrobial stewardship concerns, and its use in the community setting carries a risk of selecting for multidrug-resistant strains. Faropenem is structurally related to carbapenems, and preliminary in vitro evidence suggests that faropenem resistance may foster cross-resistance to carbapenems, although clinical data remain limited [10]. Given rising antimicrobial resistance in Bangladesh and similar settings, indiscriminate faropenem use could compromise carbapenem effectiveness for severe infections. Faropenem should therefore be reserved for patients with risk factors for multidrug-resistant infection, such as documented or probable resistance to first-line agents, recent broad-spectrum antibiotic exposure or healthcare contact, or clinical failure on a narrower regimen, with prescribing controlled and guided by local antibiograms and surveillance of penem and carbapenem susceptibility [10]. This trial, however, enrolled unselected patients and did not assess risk factors for multidrug-resistant infection, so it provides no direct evidence for faropenem in these patients. Future trials should include baseline and post-treatment microbiological sampling and assessment of gut colonization with carbapenemase-producing Enterobacterales. Non-inferiority on a clinical endpoint does not position faropenem ahead of the narrower-spectrum oral agents recommended for first-line therapy [4].

Strengths and limitations

Strengths of this study include its randomized, multicenter design, well-balanced baseline characteristics, complete follow-up with no attrition, use of an active comparator recommended for adults with comorbidity [4,6], reporting in accordance with the CONSORT statement [11], and presentation of absolute and relative treatment effects with CIs rather than p-values alone.

Several limitations should be acknowledged. First, the open-label design and investigator-defined, unvalidated, improvement-based primary endpoint are important limitations. Because neither participants nor assessing physicians were masked, assessment bias cannot be excluded for clinical cure, day 3 cure, and adverse event ascertainment. The threshold itself was applied identically in both arms and is therefore unlikely to have favored faropenem, but it may also have classified participants with persistent symptoms as cured, inflating cure rates in both arms and biasing the non-inferiority comparison toward similarity; these rates are therefore not directly comparable with those from trials using resolution-based definitions. Future trials should use blinded or independently adjudicated assessment with a validated cure definition.

Second, no microbiological or etiologic diagnosis was performed. Consequently, clinical cure could not be linked to specific pathogens; the contribution of atypical pathogens, against which faropenem lacks activity, is unknown; microbiological equivalence between regimens remains unproven; and whether broad-spectrum therapy was microbiologically warranted for individual participants cannot be determined. This is especially important because the trial replaced beta-lactam-macrolide combination therapy with beta-lactam monotherapy. Given Bangladesh’s antimicrobial resistance burden [2,10], unnecessary penem exposure carries a correspondingly greater ecological cost.

Third, the modest sample size was calculated to assess non-inferiority, limiting the study's ability to detect differences in efficacy, conduct subgroup analyses, and identify uncommon adverse events. The calculation assumed a 9.9-percentage-point faropenem advantage using cure rates from separate trials that differed in populations, endpoint definitions, comparators, and faropenem formulations; had the true rates been equal, the trial would have been underpowered.

Fourth, allocation concealment was imperfect because investigators enrolled participants and assigned treatments using fixed blocks of four, making the final allocation within each block potentially predictable. Sequentially numbered, sealed envelopes reduced but may not have eliminated this risk.

Fifth, investigators limited enrollment to mild-to-moderate disease, assessed clinically and radiologically rather than with CURB-65 or the Pneumonia Severity Index, and did not record baseline oxygen saturation. Thus, the population cannot be characterized precisely, and findings cannot be extrapolated to severe or hospitalized CAP. The comparator was uniform across the trial and, for the approximately one-third of participants without comorbidity, broader in spectrum than the monotherapy recommended for that group [4]. Although a broader comparator makes non-inferiority harder rather than easier to demonstrate, the study was not powered to compare faropenem with guideline-concordant monotherapy in this subgroup.

Finally, follow-up was limited to clinical cure at days 10-14 and safety through day 28, precluding assessment of late relapse and delayed adverse effects. As the trial was conducted in one country, the findings, including the resistance and stewardship considerations, may not generalize to other populations or health systems.

## Conclusions

In this open-label trial of adults with mild-to-moderate CAP who did not require hospitalization and had no significant renal or hepatic impairment, oral faropenem was non-inferior to a standard regimen of co-amoxiclav plus clarithromycin for clinical cure, with a comparable early clinical response and a comparable safety profile. Most participants in both arms achieved clinical cure, with no serious adverse events or treatment withdrawals. Hepatic and renal function remained stable in both groups over the study period.

These findings suggest that faropenem monotherapy may offer an effective, well-tolerated oral option for patients for whom a narrower-spectrum first-line regimen is unsuitable, replacing a two-drug regimen with a single agent, although larger, blinded trials with microbiologically characterized populations are needed to confirm these findings. Because no microbiological diagnosis was undertaken, these results describe clinical outcomes only and cannot establish pathogen-specific efficacy.
